# Assessment of Patient-Preferred Language to Achieve Goal-Aligned Deprescribing in Older Adults

**DOI:** 10.1001/jamanetworkopen.2021.2633

**Published:** 2021-04-05

**Authors:** Ariel R. Green, Hélène Aschmann, Cynthia M. Boyd, Nancy Schoenborn

**Affiliations:** 1 Division of Geriatric Medicine and Gerontology, Johns Hopkins University School of Medicine, Baltimore, Maryland; 2 Epidemiology, Biostatistics and Prevention Institute, University of Zurich, Zurich, Switzerland; 3 Department of Health Policy and Management, Johns Hopkins University Bloomberg School of Public Health, Baltimore, Maryland

## Abstract

**Importance:**

How clinicians communicate about deprescribing, the structured process of reducing or stopping unnecessary, potentially harmful, or goal-discordant medicines, may be associated with the extent to which older adults are willing to do it.

**Objective:**

To examine older adults’ preferences regarding different rationales a clinician may use to explain why a patient should stop an unnecessary or potentially harmful medication.

**Design, Setting, and Participants:**

This cross-sectional survey study was conducted from March 25 to April 19, 2020, among a nationally representative, probability-based online survey panel (KnowledgePanel). KnowledgePanel members aged 65 years and older were recruited by random digit dialing and address-based sampling. Data were analyzed from May 4 to July 8, 2020.

**Exposures:**

The survey presented 2 vignettes involving hypothetical older adults. One described a statin being used for primary prevention by a person with functional impairment and polypharmacy. The second described a sedative-hypnotic, such as zolpidem, being used for insomnia by a person with good functional status.

**Main Outcomes and Measures:**

After each vignette, participants expressed preferences using a best-worst scaling method for 7 different phrases a clinician may use to explain why they should reduce or stop the medication. Conditional logistic regression was used to quantify respondents’ relative preferences.

**Results:**

A total of 1193 KnowledgePanel members were invited, and 835 respondents (70.0%) completed the survey. The mean (SD) age was 73 (6) years, 414 (49.6%) were women, and 671 (80.4%) self-identified as White individuals. A total of 496 respondents (59.8%) had ever used a statin, and 124 respondents (14.9%) had ever used a sedative-hypnotic. For both medications, the most preferred phrase to explain deprescribing focused on the risk of side effects. For statins, this phrase was 5.8-fold (95% CI, 5.3-6.3) more preferred than the least preferred option, which focused on the effort (treatment burden) involved in taking the medicine. For sedative-hypnotics, the phrase about side effects was 8.6-fold (95% CI, 7.9-9.5) more preferred over the least preferred option, “This medicine is unlikely to help you function better.”

**Conclusions and Relevance:**

These findings suggest that among older adults, the most preferred rationale for deprescribing both preventive and symptom-relief medicines focused on the risk of side effects. These results could be used to inform clinical practice and improve effective communications around deprescribing in older adults.

## Key Points

**Question:**

What rationale for deprescribing, the structured process of reducing or stopping unnecessary, potentially harmful, or goal-discordant medicines, do older adults prefer for clinicians to use?

**Findings:**

In this survey study of 835 older adults, respondents’ preferred phrases to explain deprescribing preventive and symptom-relief medicines focused on the risk of side effects. For preventive medicines, other more preferred phrases included references to using a harmful total number of medicines and benefits not outweighing risks, and for symptom-relief medicines, other more preferred phrases included references to working together to slowly reduce the dose and the medicine causing more harm than good.

**Meaning:**

These findings suggest that for deprescribing to succeed, it requires effective communication that resonates with patients.

## Teaser

This survey study examines older adults’ preferences regarding different rationales a clinician may use to explain why a patient should stop an unnecessary or potentially harmful medication.

## Introduction

Polypharmacy is associated with adverse drug events and harmful effects on quality of life and health outcomes.^1,2^ Older adults are especially at risk of polypharmacy. Deprescribing, the structured process of reducing or stopping unnecessary, potentially harmful, or goal-discordant medicines, is an important and underused approach to minimizing iatrogenic harm for older adults.^3^ There is insufficient evidence on how to best achieve deprescribing in older adults, although a growing body of research supports patient- and family caregiver–centered approaches.^4^

How clinicians communicate about deprescribing may affect to what extent patients and caregivers understand and participate in it. When patients are fully informed about the risks and benefits of available treatments and engaged in decision-making, they are more likely to choose more conservative treatment approaches, including deprescribing.^5,6^ Existing literature has focused on factors weighed by patients when deciding whether to use a medicine. To our knowledge, no prior study has examined how patients perceive various rationales a clinician may give to explain reducing or stopping a medicine that the patient is already using. If certain characteristics of a medication are more likely to motivate a patient to stop using it, in the context of the clinician believing that deprescribing is in the best interest of the patient and is aligned with the patient’s goals of care, deprescribing communication would ideally emphasize those characteristics. If clinician communication regarding deprescribing does not resonate with patients and caregivers, deprescribing is not likely to succeed.

The objective of this national survey was to examine older adults’ preferences regarding different phrases that a clinician may use to explain why a patient should stop an unnecessary or potentially harmful or medication. We examined separately 2 hypothetical scenarios. The first was a statin being used for primary prevention by a patient with multimorbidity, functional impairment, and polypharmacy, considering that there is substantial uncertainty regarding the balance of benefits and harms of statins for primary prevention in such patients, and it is a preference-sensitive decision.^7,8^ The second was a benzodiazepine receptor agonist being used for insomnia, a bothersome but not life-threatening condition, by a fairly healthy older adult for whom the decision of whether or not to use a sedative-hypnotic may not be straightforward. Although sedative-hypnotics are generally contraindicated in all older adults,^9^ the recommendation to avoid such medications is more clear in older adults with mobility impairments and cognitive deficits, in whom they pose the highest risk of serious adverse effects, whereas the benefit-to-harm ratio is less clear in healthy older adults.^10^ We focused on scenarios in which the clinician, in the context of a trusting relationship and based on knowledge of the patient’s health and goals of care, believes it is in the patient’s best interest to stop the medicine.

## Methods

This study was approved by the Johns Hopkins University School of Medicine institutional review board and followed the American Association for Public Opinion Research (AAPOR) reporting guideline. The beginning of the survey stated that completion of the survey served as consent to be in the research study. Additional details about Ipsos KnowledgePanel are available in eMethods in the Supplement.

### Study Design and Sample

We conducted a cross-sectional survey study of individuals aged 65 years and older from KnowledgePanel (Ipsos), an online research panel with approximately 60 000 members that is designed to be representative of US adult population (eTable 1 in the Supplement). Panel members were randomly recruited by random digit dialing (until 2009) and address-based sampling (since 2009).^11^ Households without computers or internet access were provided with both. The target population consisted of noninstitutionalized adults aged 65 years and older in the United States.

### Survey Instrument

The survey was designed to test participants’ preferences for 7 different rationales that a clinician may use to explain why a patient should reduce or stop an unnecessary or potentially harmful medication (Box; eMethods in the Supplement). We separately examined 2 hypothetical scenarios, one in which a statin was being used for primary prevention (module 1), and one in which a sedative-hypnotic in the benzodiazepine receptor agonist class, zolpidem, was being used for insomnia (module 2). Each participant completed both modules in the same order. The hypothetical scenarios were developed by the study team, which included 3 geriatricians, and revised based on pilot testing with older adults. The phrases (ie, the language used to explain those rationales) were identified from previous qualitative research involving older adults, family caregivers, and physicians^12,13^ and literature review. In the statin module, our goal was to describe an older adult with multiple chronic conditions, functional impairment, and polypharmacy. We described a woman aged 78 years with multiple chronic conditions (ie, oxygen-dependent pulmonary disease, arthritis, hypertension, and mild cognitive impairment), functional impairment (ie, “needs her daughter’s help to leave the house”), and polypharmacy (ie, “10 pills per day”) who uses a statin for primary prevention. Statins were described as a class of medicine that lowers risk of heart attacks and strokes. We also described the risks: muscle pain or weakness, nausea, constipation, diarrhea, and drug interactions. The statin module also alluded to uncertainties in the evidence (eg, “for some older people who have never had a heart attack or stroke – especially people who have several other health problems – we don’t know for sure that the benefits of cholesterol medicines outweigh the risk of side effects”). In the sedative-hypnotic module, our goal was to describe a fairly healthy older adult. We described a man aged 78 years with diabetes, hypertension, low back pain, and good functional status. He used 6 pills per day, including zolpidem. Sedative-hypnotics were described as a type of medicine that helps with a bothersome but not life-threatening symptom. Risks were described as “falls, memory problems, hospitalizations and death.”

Box. **Phrases Tested**

**Explanations Clinicians May Use to Explain Why Someone Should Reduce or Stop a Statin Medicine**

“The benefits of this medicine do not clearly outweigh the risks for people like you.”
“I do not feel that you need this medicine anymore.”
“Given your age and other health problems, I do not think this medicine will help you.”
“Given your age and other health problems, I’m worried that you are at increased risk of side effects from this medicine.”
“Taking this medicine requires extra effort for you. It’s another pill to swallow, costs you money, and requires periodic blood tests.”
“I think it could be harmful for you to be on this many medicines.”
“I think we should focus on how you feel now rather than thinking about things that might happen years down the road.”

**Explanations Clinicians May Use to Explain Why Someone Should Reduce or Stop a Sedative-Hypnotic**

“I’m worried that this medicine may cause you more harm than good.”
“Medical guidelines recommend that we avoid prescribing this medicine for sleeping problems in older adults.”
“Over the long run, this medicine is unlikely to help you function better.”
“This medicine has been linked to side effects such as problems with memory, concentration, balance and falls, hospitalizations and death in older adults.”
“People can become dependent on this medicine, meaning that they cannot fall asleep without it.”
“We can treat this condition without medicine. It will take time and effort, but you can learn to fall asleep on your own.”
“This medicine is not good for you in the long run; let’s work together to slowly reduce the dose and get you off it over time.”

We designed the survey using the best-worst scaling method, which systematically measures stated preferences of respondents. In a series of best-worst scaling tasks, respondents were presented with 3 phrases at a time and asked to choose the best and the worst phrase.^14^ The survey for each medication class consisted of 7 choice tasks, in which we used the balanced incomplete block design (generated using SAS statistical software version 9.4 [SAS Institute]) to systematically vary the phrases presented in each choice task. Best-worst scaling decreases cognitive burden placed on respondents by asking to compare only a few options at a time instead of all at once. The survey also collected information on demographic characteristics, overall health, health literacy,^15^ and general attitudes and experiences relating to medicines. A final section asked participants to rate their level of agreement with 3 statements adapted from prior studies on health outcome prioritization among older adults with multiple chronic conditions.^16,17^ The statements focused on trade-offs between quantity and quality of life and between current and future health. We pilot tested the survey instrument with 9 older adults who were not included in the study and iteratively revised it based on feedback.

### Statistical Analysis

The main analysis used conditional logit regression. A phrase was assigned a value of −1 if it was chosen as the least preferred phrase and +1 if it was the most preferred phrase, while accounting for clustering by respondent and by choice task. The regression coefficients for each phrase measure the respondents’ preference for that phrase relative to the other phrases on an odds ratio scale in which we set the least preferred phrase as the reference, so that a phrase with a coefficient of 2 means that the odds of choosing this phrase as the most preferred one were 2-fold as large compared with the reference.

To assess the variability of preferences, we calculated individual best-minus-worst scores. Best-minus-worst scores count how many times a phrase was selected as best (most preferred) or worst (least preferred). The distribution of scores was −3 to 3, as each phrase appeared in 3 of 7 choice tasks. Furthermore, to explore potential associations of preferences with respondent characteristics, we performed preplanned (hypothesis-driven) linear regressions of individual best-minus-worst scores in which each phrase was tested in a separate model with age, overall number of medications, prior use of statins or sedative-hypnotics, self-reported health, and health literacy as the variables of interest. For sedative-hypnotics, we also tested a combined variable of falls in the past year and memory concerns as a variable of interest. We did not do formal hypothesis testing, but highlighted baseline characteristics that were associated with preferences for phrases to explain deprescribing at an unadjusted, 2-sided significance level of *P* = .01 (eTable 2 in the Supplement). All analyses were performed using R statistical software version 3.6.3 (R Project for Statistical Computing). Data were analyzed from May 4 to July 8, 2020.

## Results

### Respondent Characteristics

Among 1193 eligible panel members invited to participate, 836 (70.1%) completed the survey between March 25 and April 19, 2020. We excluded 1 respondent who did not answer any questions, yielding a total analytic sample of 835 respondents. The mean (SD) age of respondents was 73 (6) years; 414 (49.6%) were women, and 671 (80.4%) self-identified as White individuals (Table 1). In addition, 216 respondents (26.0%) reported concerns about their memory, and 145 respondents (17.4%) ranked their health as fair or poor. A total of 496 respondents (59.8%) had ever used a statin, and 124 respondents (14.9%) had ever used a sedative-hypnotic (Table 2). In the sedative-hypnotic module, 25 respondents (2.9%) had at least 1 missing response (either the most or least preferred option). In the statin module, 54 respondents (6.5%) had at least 1 missing response. There was moderate to high intrarespondent consistency of responses across all tasks (eFigure in the Supplement).

**Table 1.** Sociodemographic Characteristics of Study Participants

Characteristic	No. (%) (N = 835)^**a**^
Age, mean (SD), y	73 (6)
Sex	
Women	414 (49.6)
Men	421 (50.4)
Race/ethnicity	
White, non-Hispanic	671 (80.4)
African American, non-Hispanic	61 (7.3)
Hispanic	58 (6.9)
≥1 race or other^b^	45 (5.4)
Educational level	
Did not complete high school	46 (5.5)
Completed high school	252 (30.2)
<4 y college	232 (27.8)
College graduate or postgraduate degree	305 (36.5)
Confidence filling out medical forms	
Extremely	558 (67.1)
Quite a bit	174 (20.9)
Somewhat	69 (8.3)
A little bit	20 (2.4)
Not at all	11 (1.3)
Difficulty paying for medicines	
Extremely	10 (1.2)
Somewhat	80 (9.6)
Not at all	706 (84.6)
Unsure	16 (1.9)
Do not wish to answer	19 (2.3)

^a^ The number of respondents varied between 831 and 835, given that some respondents did not answer all questions.

^b^ Includes American Indian or Alaska Native, Native Hawaiian or Pacific Islander, or Asian.

**Table 2.** Health Status of 835 Study Participants

Characteristic	No. (%) (N = 835)^**a**^
Total medicines per d, mean (SD), No.	4.1 (3.3)
Self-rated health status	
Excellent	64 (7.7)
Very good	296 (35.5)
Good	328 (39.3)
Fair	127 (15.2)
Poor	18 (2.2)
Ever used a statin	496 (59.8)
Ever used a prescription sleep aid	124 (14.9)
History of falls in the past y	215 (25.8)
Self-reported concerns about memory	216 (26.0)
Has ever had a heart attack, coronary artery disease, or angina	135 (16.2)
Has ever had a stroke	55 (6.6)
Has ever had diabetes	167 (20.0)
Has insomnia on a regular basis	202 (24.3)
Would be willing to stop a statin if doctor recommended it	507 (60.9)
Would be willing to stop a medicine for a bothersome but not life-threatening symptom if doctor recommended it	419 (50.3)
Has ever had emergency department visit or hospitalization owing to medication side effects	37 (4.4)
Has ever had bad experience due to stopping a medicine	58 (7.0)

^a^ The number of respondents varied between 829 and 835, given that some respondents did not answer all questions.

### Ranking of Phrases

**Figure 1.**
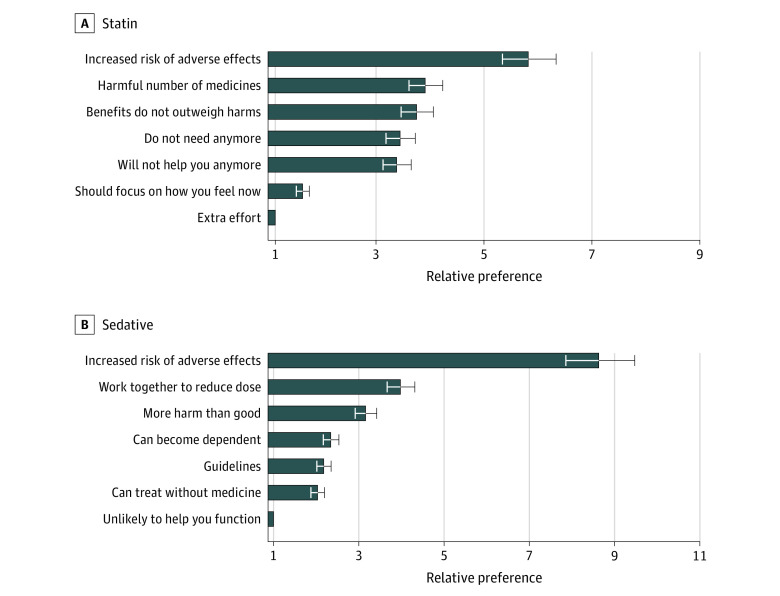
Relative Preference of 835 Older Adults Regarding 7 Phrases a Clinician May Use to Explain Deprescribing A phrase was assigned a value of −1 each time it was chosen as the least preferred phrase and +1 if it was the most preferred phrase, while accounting for clustering by respondent and by choice task. We set the least preferred phrase as the reference, so that a phrase with a preference weight of 2 is twice as preferred relative to the reference phrase.

The most preferred phrase to explain reducing or stopping a statin was “Given your age and other health problems, I’m worried that you are at increased risk of side effects from this medicine.” This phrase was 5.8-fold (95% CI, 5.3 to 6.3) more preferred than the least preferred option, “Taking this medicine requires extra effort for you. It’s another pill to swallow, costs you money, and requires periodic blood tests” (Figure 1A; eTable 3 in the Supplement). Other more preferred phrases included references to using a harmful total number of medicines, benefits not clearly outweighing risks, the patient not needing the medicine anymore, and “Given your age and other health problems, I do not think this medicine will help you.” Another less preferred phrase was “I think we should focus on how you feel now rather than thinking about things that might happen years down the road.”

As with statins, the most preferred phrase to explain reducing or stopping a sedative-hypnotic concerned side effects. This phrase was 8.6-fold (95% CI, 7.9 to 9.5) more preferred vs the least preferred option, “Over the long run, this medicine is unlikely to help you function better” (Figure 1B; eTable 3 in the Supplement). Other more preferred phrases included “This medicine is not good for you in the long run; let’s work together to slowly reduce the dose and get you off it over time,” and a reference to the medicine causing the patient “more harm than good.” Less preferred phrases focused on the risk of dependency, use of nonpharmacologic options, and guidelines.

### Variability of Preferences Between Individuals

**Figure 2.**
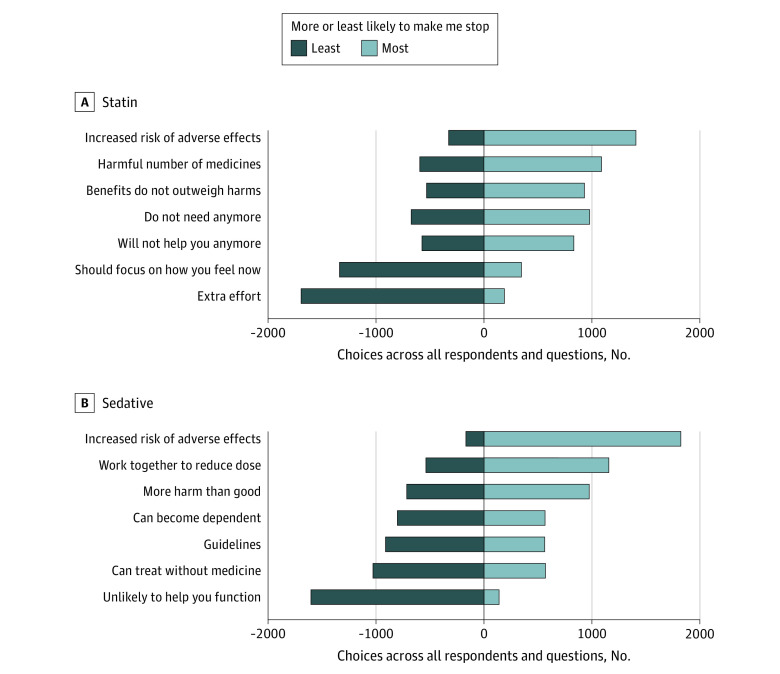
Variability of Preferences Between Individuals The figure shows the number of times each explanation was chosen as most and as least likely to make a participant stop the medicine, ranked from most likely (right) to least likely (left). Each explanation was presented 3 times to each participant, so the maximum number of times it could be chosen as most or least likely was 2505.

No single phrase was always chosen as the most or least preferred. For statins, 214 respondents (25.6%) always chose the phrase about risk of side effects as the most likely to make them stop a statin across all 7 choice tasks, while 381 respondents (45.6%) always chose the phrase about extra effort as least likely to make them stop using a statin. For sedative-hypnotics, 413 respondents (49.5%) always chose risk of side effects as the most likely to make them stop using a sedative-hypnotic, while 268 respondents (32.1%) always chose the phrase about lack of improved functioning as least likely to make them stop a sedative-hypnotic. Figure 2 shows the number of times each explanation was chosen as most and as least likely to make a participant stop the medicine.

In linear regressions of individual best-minus-worst scores with respondent characteristics, respondents who had used a prescription sleep medicine were significantly less likely than respondents who had not used one to prefer the rationale about guidelines (individual best-minus-worst scores, −0.51 [95% CI, −0.82 to −0.20] points lower on a scale from −3 to 3 points). Older respondents were less likely than younger ones to prefer the explanation that a sedative-hypnotic could cause “more harm than good” (individual best-minus-worst scores, −0.13 [95% CI, −0.21 to −0.05] points). Respondents with falls or memory concerns were less likely than respondents without falls or memory concerns to prefer the phrase about nonpharmacologic treatments (individual best-minus-worst scores, −0.35 [95% CI −0.59 to −0.11] points). For statins, respondents with higher self-reported health literacy were less likely to prefer the phrase focusing on effort than those with low to moderate health literacy (individual best-minus-worst scores, −0.50 [95% CI, −0.83 to −0.17] points) (eTable 2 in the Supplement).

## Discussion

In this national survey study, we characterized older adults’ preferences for how clinicians can discuss deprescribing potentially inappropriate medications. We found that the most preferred explanation for deprescribing statins and sedative-hypnotics focused on the risk of side effects. To our knowledge, this is the first study to quantify older adults’ perspectives on deprescribing communication. This is important because how clinicians introduce deprescribing during clinical encounters may affect to what extent patients understand and accept it.

Our findings concerning statins were consistent with results of earlier studies investigating patients’ priorities regarding treatment decisions, which showed that among multimorbid older adults, the factor most associated with willingness to take a preventive medication was the type and severity of adverse effects associated with the medicine, rather than the degree of benefit to be gained from it.^18,19^ Taken together with this prior research,^18,19^ our results suggest that phrases, such as the one we tested “Given your age and other health problems, I’m worried that you are at increased risk of side effects from this medicine,” may be effective ways to frame deprescribing. The earlier studies^18,19^ examined how older adults’ willingness to use a preventive medication was associated with varying benefits and harms. Our study adds to this literature by investigating how older adults want clinicians to communicate about stopping medications that they are already using, including symptom-relief medications, and by investigating various rationales for deprescribing. We found that explanations centered on the extra effort involved in taking medicines and prioritizing how a patient feels now over long-term mortality benefits ranked lower than the phrase that mentioned side effects. This is in line with results of a 2014 study^20^ suggesting that treatment burden was not a factor strongly associated with treatment preference for noninsulin type 2 diabetes medications. If patients believe their medications are necessary, as most older adults do,^21^ they may be willing to go to great lengths to use them. The result also indicates that older adults or their families may react negatively to deprescribing language that conveys they no longer stand to benefit from prevention. A recommendation to deintensify preventive therapies may be perceived as rationing care based on age or life expectancy if it is not framed it as a way of preserving well-being.^22^

For both drug classes, we found substantial variability in respondents’ preferences, implying that the preferred approach may not be the same for all patients. For example, language focused on deintensification to reduce treatment burden and prioritize quality of life may resonate with seriously ill individuals or their family caregivers.^12^ Although the phrase about extra effort, or treatment burden, involved in taking a statin was least preferred overall, some respondents ranked this phrase as most likely to make them stop a statin. Clinicians need to understand the patient’s or caregiver’s priorities so that they can individualize deprescribing recommendations and tailor their language accordingly.^23^ A structured, facilitator-led approach in primary care that aligns clinical decision-making with patients’ goals and preferences may reduce treatment burden and unwanted health care, including medications, for older adults with multiple comorbidities.^24^ For example, it may be effective for clinicians to frame deprescribing recommendations around an individual’s goals: “Because your goal is to be able to walk your granddaughter to school, I think this medicine could be harmful for you.” Future studies should determine how best to implement such an approach to deprescribing in routine clinical care. While we found associations between communication preferences and some respondent characteristics at an unadjusted significance level of *P* = .01, considering the multitude of characteristics and phrases we analyzed, there is uncertainty in these associations. Importantly, these patient characteristics did not explain much of the variation in preferences, which suggests that patients cannot be assumed to have particular preferences based on age, health status, or other factors.

With sedative-hypnotics, respondents again preferred framing that emphasized the risk of side effects. This was strongly preferred over explanations concerning improved functioning or those based on the risk of dependency, the use of nonpharmacologic options, and guidelines. This finding highlights the importance of informing patients and caregivers about risks, such as falls and cognitive impairment associated with sedative-hypnotics, since they may not be aware of these potential harms. Previous research suggests these adverse effects are rarely discussed in current practice, even for older adults who have already experienced them.^25,26^ For example, dementia caregivers often believe antipsychotic medications are safe despite their well-documented potential for adverse effects.^27^ It is possible that the phrase about nonpharmacologic options (ie, “You can learn to fall asleep on your own”) was ranked low because of outside influences, such as direct-to-consumer prescription drug advertising, which tends to emphasize benefits and downplay risks,^28^ although we did not explore the reasons underlying respondents’ preferences.

With respect to sedative-hypnotics, another highly ranked phrase was “This medicine is not good for you in the long run; let’s work together to slowly reduce the dose and get you off it over time.” This phrase may have been preferred by many respondents because it conveys that deprescribing will be a shared decision between the patient and doctor and the change will be made gradually. This echoes results of 2020 qualitative research from Green et al^12^ that reported on the importance of establishing trust before deprescribing and providing close follow-up to address recurrence of symptoms.

### Limitations

Our study has several limitations. First, the survey used hypothetical scenarios, which may not reflect the choices participants would actually make. Second, we compared individual phrases, although clinicians are likely to use multiple phrases when talking with patients. Third, our sample may not be representative of older adults with serious illness, cognitive impairment, or low health literacy. However, 26% of respondents reported concerns about their memory, and 17% ranked their health as fair or poor. Fourth, the best-worst scaling method may be confusing to participants. However, most tasks (93% for statins and 97% for sedative-hypnotics) were filled out completely, and there was high consistency, suggesting that the survey was well understood. Fifth, the study focused on patient-clinician communication only, although other health care professionals, such as clinical pharmacists and nurses, may play an important role in deprescribing communication with patients. Sixth, we tried to minimize respondent burden by keeping the survey under 20 minutes, though it may have strengthened our conclusions to study additional scenarios.

## Conclusions

These findings suggest that although most older adults and caregivers may be willing to have a medication deprescribed if their doctor says it is possible,^21^ uptake of deprescribing has not been widespread. Patient involvement is key, and deprescribing must be framed in language that is acceptable to patients. We found that the most preferred rationale for deprescribing centered on the risk of side effects. Explanations focused on the extra effort involved in taking a preventive medication or the lack of functional benefit for sedative-hypnotics were least preferred. Our findings highlight the importance of linking deprescribing to the patient’s medical history or concerns about falls and memory impairment, introducing it in language that conveys the shared nature of the decision, and framing it as a positive step to preserve well-being, not a withdrawal of care based on age or life expectancy.

## Notes

Supplement. **eMethods.** Supplementary Methods **eTable 1.** Comparison of Survey Respondents and US Population Benchmarks Among Adults Aged 65 Years and Older **eTable 2.** Associations of Respondent Characteristics With Preferences for Phrases to Explain Deprescribing **eTable 3.** Relative Preference of Older Adults Regarding 7 Phrases a Clinician May Use to Explain Deprescribing **eFigure.** Best-Minus-Worst Score Variance
