# Supplementary material for: Assessment of Patient-Preferred Language to Achieve Goal-Aligned Deprescribing in Older Adults
Source: JAMA Netw Open. 2021 Apr 5;4(4):e212633. doi: 10.1001/jamanetworkopen.2021.2633 (PMC8022217; doi:10.1001/jamanetworkopen.2021.2633)
Supplement: Supplement. — eMethods. Supplementary Methods eTable 1. Comparison of Survey Respondents and US Population Benchmarks Among Adults Aged 65 Years and Older eTable 2. Associations of Respondent Characteristics With Preferences for Phrases to Explain Deprescribing eTable 3. Relative Preference of Older Adults Regarding 7 Phrases a Clinician May Use to Explain Deprescribing eFigure. Best-Minus-Worst Score Variance [file jamanetwopen-e212633-s001.pdf]

Supplemental Online Content

Green AR, Aschmann H, Boyd CM, Schoenborn N. Assessment of patient-preferred language to achieve goal-aligned deprescribing in older adults. *JAMA Netw Open*. 2021;4(4):e212633. doi:10.1001/jamanetworkopen.2021.2633

eMethods. Supplementary Methods
eTable 1. Comparison of Survey Respondents and US Population Benchmarks Among Adults Aged 65 Years and Older
eTable 2. Associations of Respondent Characteristics With Preferences for Phrases to Explain Deprescribing
eTable 3. Relative Preference of Older Adults Regarding 7 Phrases a Clinician May Use to Explain Deprescribing
eFigure. Best-Minus-Worst Score Variance

This supplemental material has been provided by the authors to give readers additional information about their work.

© 2021 Green AR et al. *JAMA Network Open.*

eMethods. Supplementary Methods

DEPRESCRIBING COMMUNICATION SURVEY

Understanding older adults' preferences about medication communication

Researchers at Johns Hopkins University designed this survey. The survey tries to understand how people think about their medications and how they want their doctors to talk with them about reducing or stopping medications. We hope this information will help doctors improve how they communicate with older adults.

The survey has 3 sections and takes about 25 minutes. There are no right or wrong answers. We want your honest opinions and feelings. Your completion of this survey will serve as your consent to be in this research study. Your answers will not be traced back to you.

© 2021 Green AR et al. *JAMA Network Open.*

Section 1

Has a doctor ever told you that you have had a heart attack, coronary artery disease, or angina?

☐ Yes
☐ No

Has a doctor ever told you that you have had a stroke?

☐ Yes
☐ No

Has a doctor ever told you that you have diabetes?

☐ Yes
☐ No

Have you ever required treatment for anxiety or depression?

☐ Yes
☐ No

Do you have insomnia (difficulty falling asleep or staying asleep) on a regular basis?

☐ Yes
☐ No

During the past 12 months, how many times have you been hospitalized overnight?

☐ Zero
☐ One
☐ Two or more

How many different medicines per day are you taking now (for all of your health needs)? Please write your answer on the line below.

______

How do you feel about the number of medicines you take?

☐ Just right
☐ Too many
☐ Think you need a medicine that your doctor is *not* currently prescribing

Have you ever had an Emergency Department visit or hospitalization related to side effects from taking a medicine?

☐ Yes
☐ No

Have you ever had a bad experience because you or your doctor reduced or stopped a medicine?

☐ Yes
☐ No

Have you ever taken a prescription medicine to help you sleep? Common sleeping pills include zolpidem (sold as Ambien), eszopiclone (sold as Lunesta), alprazolam (sold as Xanax) and clonazepam (sold as Klonopin).

☐ Yes
☐ No

Have you ever taken a prescription medicine to lower your cholesterol, such as atorvastatin (sold as Lipitor), simvastatin (sold as Zocor) or pravastatin (sold as Pravachol)?

☐ Yes
☐ No

© 2021 Green AR et al. *JAMA Network Open.*

Have you fallen at least once in the past year?
☐ Yes
☐ No

Do you have any concerns about your memory?
☐ Yes
☐ No

In general, would you say your health is… (*Select one answer only.*)
☐ Excellent
☐ Very good
☐ Good
☐ Fair
☐ Poor

How confident are you filling out medical forms by yourself? (*Select one answer only.*)
☐ Extremely
☐ Quite a bit
☐ Somewhat
☐ A little bit
☐ Not at all

How difficult is it for you (and your family) to pay for your medicines on a monthly basis? (*Select one answer only.*)
☐ Extremely difficult
☐ Somewhat difficult
☐ Not at all difficult
☐ Unsure
☐ Do not wish to answer

Section 2
Medicine to lower risk of future health problems:
Many people take **statins**, which are medicines used to lower cholesterol. Too much cholesterol in the blood can clog your arteries, increasing risk of future heart attacks and strokes. **Even if you do not take a statin, we are interested in your opinion.**

However, for some older people who have never had a heart attack or stroke – especially people who have several other health problems – we don't know for sure that the benefits of cholesterol medicines outweigh the risk of side effects.

All medicines have potential side effects. Although statins are usually well-tolerated, some older adults are at higher risk of statin side effects such as muscle pain or weakness, nausea, constipation or diarrhea. Many older people take a lot of medicines. Other medicines can interact with statins and lead to serious problems.

Sometimes, doctors may recommend reducing or stopping statins in older adults because the potential harms of the statins outweigh the potential benefits.

Please answer the following question:
If your doctor recommended that you **stop** taking a medicine, such as a statin, that could lower your risk of future health problems, such as heart attacks and strokes, but may cause side effects, would you be willing?
☐ Yes
☐ No
☐ Maybe

Now we would like to know your opinion about how doctors can better explain why a person should stop a statin. Below are the different explanations the doctor could give. **This is just an example; you do not have to fill anything out in this table.**

© 2021 Green AR et al. *JAMA Network Open.*

Explanations doctors may use to explain why someone should reduce or stop a statin medicine
The benefits of this medicine do not clearly outweigh the risks for people like you.
I do not feel that you need this medicine anymore.
Given your age and other health problems, I do not think this medicine will help you.
Given your age and other health problems, I'm worried that you are at increased risk of side effects from this medicine.
Taking this medicine requires extra effort for you. It's another pill to swallow, costs you money, and requires periodic blood tests.
I think it could be harmful for you to be on this many medicines.
I think we should focus on how you feel now rather than thinking about things that might happen years down the road.

Imagine that Ms. Brown is 78 years old. She has lung disease requiring that she wear oxygen, pain in her joints due to arthritis, and needs her daughter's help to leave the house. She also has high blood pressure and mild memory problems. She takes 10 pills per day. Ms. Brown is at a doctor's appointment. She has seen this doctor a couple of times before and she trusts her. The doctor thinks that Ms. Brown should stop taking the statin medicine, atorvastatin (also called Lipitor), that she has been taking. Ms. Brown has never had a heart attack or stroke and does not have diabetes. She does not think that she has experienced any side effects from the statin.

We want to know, if you were Ms. Brown, which explanations would make you most likely to stop the atorvastatin?

In each task, we will show you 3 explanations that the doctor could give for stopping the atorvastatin. Within each block of 3 explanations, please pick the **one explanation most likely to make you stop** the medicine and the **one explanation least likely to make you stop the medicine**. The explanations will be repeated in different combinations. Please complete **all** the tasks as best as you can, even if they seem repetitive. There are no right or wrong answers. Even if you don't like any of the choices, tell us which is the most likely to make you stop and the least likely to make you stop.

Example: Mary was shown the following task. **This is just an example; you do not have to fill anything out in this table.**

Most likely to make you stop	Explanations doctors could use to explain the reasons for reducing or stopping a medicine	Least likely to make you stop
☐	I do not feel that you need this medicine anymore.	☒
☒	The benefits of this medicine do not clearly outweigh the risks for people like you.	☐
☐	Given your other health problems, I do not feel that this medicine will help you.	☐

Mary carefully considered all three explanations. She chose "The benefits of this medicine do not clearly outweigh the risks for people like you" as the **one** explanation **most** likely to make her stop or reduce the medicine. She chose "I do not feel that you need this medicine anymore" as the **one** explanation **least** likely to make her stop.

Now it is your turn. You will be shown 7 tasks.

© 2021 Green AR et al. *JAMA Network Open.*

Instructions: Please pick the **one explanation most likely to make you stop** the statin medicine and the **one explanation least likely to make you stop the statin medicine**.

Most likely to make you stop	Explanations doctors could use	Least likely to make you stop
☐	Given your age and other health problems, I’m worried that you are at increased risk of side effects from this medicine.	☐
☐	Given your age and other health problems, I do not think this medicine will help you.	☐
☐	Taking this medicine requires extra effort for you. It’s another pill to swallow, costs you money, and requires periodic blood tests.	☐

© 2021 Green AR et al. *JAMA Network Open.*

Instructions: Please pick the **one explanation most likely to make you stop** the statin medicine and the **one explanation least likely to make you stop the statin medicine**.

**Most likely to make you stop**	**Explanations doctors could use**	**Least likely to make you stop**
☐	I think it could be harmful for you to be on this many medicines.	☐
☐	The benefits of this medicine do not clearly outweigh the risks for people like you.	☐
☐	Given your age and other health problems, I do not think this medicine will help you.	☐

© 2021 Green AR et al. *JAMA Network Open.*

Instructions: Please pick the **one explanation most likely to make you stop** the statin medicine and the **one explanation least likely to make you stop the statin medicine**.

**Most likely to make you stop**	**Explanations doctors could use**	**Least likely to make you stop**
☐	The benefits of this medicine do not clearly outweigh the risks for people like you.	☐
☐	Taking this medicine requires extra effort for you. It's another pill to swallow, costs you money, and requires periodic blood tests.	☐
☐	I do not feel that you need this medicine anymore.	☐

© 2021 Green AR et al. *JAMA Network Open.*

Instructions: Please pick the **one explanation most likely to make you stop** the statin medicine and the **one explanation least likely to make you stop the statin medicine**.

**Most likely to make you stop**	**Explanations doctors could use**	**Least likely to make you stop**
☐	I do not feel that you need this medicine anymore.	☐
☐	I think it could be harmful for you to be on this many medicines.	☐
☐	Given your age and other health problems, I'm worried that you are at increased risk of side effects from this medicine.	☐

© 2021 Green AR et al. *JAMA Network Open.*

Instructions: Please pick the **one explanation most likely to make you stop** the statin medicine and the **one explanation least likely to make you stop the statin medicine**.

**Most likely to make you stop**	**Explanations doctors could use**	**Least likely to make you stop**
☐	Given your age and other health problems, I do not think this medicine will help you.	☐
☐	I do not feel that you need this medicine anymore.	☐
☐	I think we should focus on how you feel now rather than thinking about things that might happen years down the road.	☐

© 2021 Green AR et al. *JAMA Network Open.*

Instructions: Please pick the **one explanation most likely to make you stop** the statin medicine and the **one explanation least likely to make you stop the statin medicine**.

**Most likely to make you stop**	**Explanations doctors could use**	**Least likely to make you stop**
☐	I think we should focus on how you feel now rather than thinking about things that might happen years down the road.	☐
☐	Given your age and other health problems, I'm worried that you are at increased risk of side effects from this medicine.	☐
☐	The benefits of this medicine do not clearly outweigh the risks for people like you.	☐

© 2021 Green AR et al. *JAMA Network Open.*

Instructions: Please pick the **one explanation most likely to make you stop** the statin medicine and the **one explanation least likely to make you stop the statin medicine**.

Most likely to make you stop	Explanations doctors could use	Least likely to make you stop
☐	Taking this medicine requires extra effort for you. It's another pill to swallow, costs you money, and requires periodic blood tests.	☐
☐	I think we should focus on how you feel now rather than thinking about things that might happen years down the road.	☐
☐	I think it could be harmful for you to be on this many medicines.	☐

© 2021 Green AR et al. *JAMA Network Open.*

Medicine to help with a bothersome (but not life-threatening) symptom:
Many people take medicines to help them sleep better. However, medicines also have side effects.

Our bodies change as we age, and some of the medicines used to help people sleep can become less helpful or even harmful because of side effects. Certain sleep medicines, such as zolpidem (also called Ambien), eszopiclone (Lunesta), alprazolam (Xanax) and clonazepam (Klonopin), may lead to problems such as falls, memory problems, hospitalizations and death for older adults.

Doctors may recommend reducing or stopping such medicines. The goal of reducing or stopping medicines is to avoid side effects and improve quality of life.

Please answer the following question:
If your doctor recommended that you **stop** taking a medicine even though it helps you with a bothersome (but not life-threatening) symptom, would you be willing?
☐ Yes
☐ No
☐ Maybe

Now we would like to know your opinion about how doctors can better explain why a person should reduce or stop a sleeping pill. Below are the different explanations the doctor could give. **This is just an example; you do not have to fill anything out in this table.**

**Explanations doctors may use to explain why someone should reduce or stop a sleeping pill**
I'm worried that this medicine may cause you more harm than good.
Medical guidelines recommend that we avoid prescribing this medicine for sleeping problems in older adults.
Over the long run, this medicine is unlikely to help you function better.
This medicine has been linked to side effects such as problems with memory, concentration, balance and falls, hospitalizations and death in older adults.
People can become dependent on this medicine, meaning that they cannot fall asleep without it.
We can treat this condition without medicine. It will take time and effort, but you can learn to fall asleep on your own.
This medicine is not good for you in the long run; let's work together to slowly reduce the dose and get you off it over time.

Imagine that Mr. Smith is 78 years old. He has diabetes, high blood pressure and low back pain. He is able to walk a block without pausing to rest. He takes 6 pills per day. He is at a doctor's appointment. He has seen this doctor a couple of times before and he trusts her. The doctor thinks that Mr. Smith should stop taking the medicine that he has been taking to help him sleep, zolpidem (also called Ambien). Mr. Smith thinks the medicine helps him sleep and does not think that he has experienced any side effects from it. The doctor will monitor his response after stopping the medicine and make changes if needed.

We want to know, if you were Mr. Smith, which explanations would make you most likely to stop the zolpidem?

In each task, we will show you 3 explanations that the doctor could give for stopping the zolpidem. Within each block of 3 explanations, please pick the **one explanation most likely to make you stop** the medicine and the **one explanation least likely to make you stop the medicine**. The explanations will be repeated in different combinations. Please complete **all** the tasks as best as you can, even if they seem repetitive. There are no right or

© 2021 Green AR et al. *JAMA Network Open.*

wrong answers. Even if you don't like any of the choices, tell us which is the most likely to make you stop and the least likely to make you stop.

Example: Mary was shown the following task. **This is just an example; you do not have to fill anything out in**

**Most likely to make you stop**	**Explanations doctors could use**	**Least likely to make you stop**
☐	This medicine has been linked to side effects such as problems with memory, concentration, balance and falls, hospitalizations and death in older adults.	☐
☐	Over the long run, this medicine is unlikely to help you function better.	☐
☐	People can become dependent on this medicine, meaning that they cannot fall asleep without it.	☐

this table.

**Most likely to make you stop**	**Explanations doctors could use to explain the reasons for reducing or stopping a medicine**	**Least likely to make you stop**
☐	Over the long run, this medicine is unlikely to help you function better.	☐
☐	We can treat this condition without medicine. It will take time and effort, but you can learn to fall asleep on your own.	☒
☒	People can become dependent on this medicine, meaning that they cannot fall asleep without it.	☐

Mary carefully considered all three explanations. She chose "People can become dependent on this medicine, meaning that they cannot fall asleep without it" as the **one** explanation **most** likely to make her stop or reduce the medicine. She chose "We can treat this condition without medicine. It will take time and effort, but you can learn to fall asleep on your own" as the **one** explanation **least** likely to make her stop.

<u>Now it is your turn. You will be shown 7 tasks.</u>

Instructions: Please pick the **<u>one explanation most likely to make you stop or reduce</u>** the zolpidem and the **<u>one explanation least likely to make you stop or reduce</u>** the zolpidem.

© 2021 Green AR et al. *JAMA Network Open.*

Instructions: Please pick the **one explanation most likely to make you stop or reduce** the zolpidem and the **one explanation least likely to make you stop or reduce** the zolpidem.

Most likely to make you stop	Explanations doctors could use	Least likely to make you stop
☐	We can treat this condition without medicine. It will take time and effort, but you can learn to fall asleep on your own.	☐
☐	I'm worried that this medicine may cause you more harm than good.	☐
☐	Over the long run, this medicine is unlikely to help you function better.	☐

© 2021 Green AR et al. *JAMA Network Open.*

Instructions: Please pick the **one explanation most likely to make you stop or reduce** the zolpidem and the **one explanation least likely to make you stop or reduce** the zolpidem.

**Most likely to make you stop**	**Explanations doctors could use**	**Least likely to make you stop**
☐	I'm worried that this medicine may cause you more harm than good.	☐
☐	People can become dependent on this medicine, meaning that they cannot fall asleep without it.	☐
☐	Medical guidelines recommend that we avoid prescribing this medicine for sleeping problems in older adults.	☐

© 2021 Green AR et al. *JAMA Network Open.*

Instructions: Please pick the **one explanation most likely to make you stop or reduce** the zolpidem and the **one explanation least likely to make you stop or reduce** the zolpidem.

Most likely to make you stop	Explanations doctors could use	Least likely to make you stop
☐	Medical guidelines recommend that we avoid prescribing this medicine for sleeping problems in older adults.	☐
☐	We can treat this condition without medicine. It will take time and effort, but you can learn to fall asleep on your own.	☐
☐	This medicine has been linked to side effects such as problems with memory, concentration, balance and falls, hospitalizations and death in older adults.	☐

© 2021 Green AR et al. *JAMA Network Open.*

Instructions: Please pick the **one explanation most likely to make you stop or reduce** the zolpidem and the **one explanation least likely to make you stop or reduce** the zolpidem.

**Most likely to make you stop**	**Explanations doctors could use**	**Least likely to make you stop**
☐	Over the long run, this medicine is unlikely to help you function better.	☐
☐	Medical guidelines recommend that we avoid prescribing this medicine for sleeping problems in older adults.	☐
☐	This medicine is not good for you in the long run; let's work together to slowly reduce the dose and get you off it over time.	☐

© 2021 Green AR et al. *JAMA Network Open.*

Instructions: Please pick the **one explanation most likely to make you stop or reduce** the zolpidem and the **one explanation least likely to make you stop or reduce** the zolpidem.

Most likely to make you stop	Explanations doctors could use	Least likely to make you stop
☐	This medicine is not good for you in the long run; let's work together to slowly reduce the dose and get you off it over time.	☐
☐	This medicine has been linked to side effects such as problems with memory, concentration, balance and falls, hospitalizations and death in older adults.	☐
☐	I'm worried that this medicine may cause you more harm than good.	☐

© 2021 Green AR et al. *JAMA Network Open.*

Instructions: Please pick the **one explanation most likely to make you stop or reduce** the zolpidem and the **one explanation least likely to make you stop or reduce** the zolpidem.

Most likely to make you stop	Explanations doctors could use	Least likely to make you stop
☐	People can become dependent on this medicine, meaning that they cannot fall asleep without it.	☐
☐	This medicine is not good for you in the long run; let's work together to slowly reduce the dose and get you off it over time.	☐
☐	We can treat this condition without medicine. It will take time and effort, but you can learn to fall asleep on your own.	☐

© 2021 Green AR et al. *JAMA Network Open.*

Section 3

Please rate **each** of the following items on a scale from 1-5.
1=Strongly Agree; 2=Agree; 3= Neither Agree nor Disagree; 4= Disagree; 5= Strongly Disagree
Please write in your rating for each item.

____ The most important thing to me is living as long as I can, no matter what kind of health I am in.
____ I am willing to accept the risk of future side effects, such as falls or memory problems, to feel better now.
____ I would prefer to take fewer medicines, even if it meant that I may not live as long or may have bothersome symptoms sometimes.

© 2021 Green AR et al. *JAMA Network Open.*

eMethods. Additional Details

From**:** Ipsos Public Affairs Project Report for the Johns Hopkins University Medicine Communications Survey. April 24, 2020.

Out of households identified through probability-based sampling, 13% expressed initial interest in joining the panel and of these 62% completed the necessary steps to become active panel members. Among the subset of active panel members who were invited for this study, the completion rate was 70%.

KnowledgePanel Methodology
KnowledgePanel is the largest online panel that relies on probability-based sampling techniques for recruitment; hence, it is the largest national sampling frame from which fully representative samples can be generated to produce statistically valid inferences for study populations. Panel members are randomly selected so that survey results can properly represent the U.S. population with a measurable level of accuracy, features that are not obtainable from nonprobability or opt-in online panels.

KnowledgePanel's recruitment process was originally based exclusively on a national Random Digit Dialing (RDD) sampling methodology. In 2009, in light of the growing proportion of cellphone-only households, Ipsos migrated to an Address Based Sampling (ABS) recruitment methodology via the U.S. Postal Service's Delivery Sequence File (DSF). ABS not only improves population coverage, but also provides a more effective means for recruiting hard-to-reach individuals, such as young adults and minorities. Households without Internet connection are provided with a web-enabled device and free internet service.

After initially accepting the invitation to join the panel, participants are asked to complete a short demographic survey (the initial Core Profile Survey); answers to this survey allow efficient panel sampling and weighting for future surveys. Upon completing the Core Profile Survey, participants become active panel members.

Survey Sampling from KnowledgePanel
Once panel members are recruited and profiled by completing the Core Profile Survey, they become eligible for selection for client surveys. Typically, specific survey samples are based on the equal probability selection method (EPSEM) for general population surveys. Customized stratified random sampling based on "profile" data can also be implemented as required by the study design.

For this particular survey, nationally representative sample of non-institutionalized U.S. adults age 65 and older was sampled.

Survey Administration
Once assigned to a survey, members receive a notification email letting them know there is a new survey available for them to complete. This email notification contains a link that sends them to the survey. No login name or password is required. The field

© 2021 Green AR et al. *JAMA Network Open.*

period depends on the client’s needs and can range anywhere from a few hours to several weeks.

Typically, after three days, automatic email reminders are sent to all non-responding panel members in the sample. Additional email reminders are sent or custom reminder schedules are set up as needed. To assist panel members with their survey taking, each individual has a personalized member portal listing all assigned surveys that have yet to be completed.

Ipsos also operates an ongoing modest incentive program to encourage participation and create member loyalty. The incentive program includes special raffles and sweepstakes with both cash rewards and other prizes to be won. Typically, we assign panel members no more than one survey per week. On average, panel members complete two to three surveys per month with durations of 10 to 15 minutes per survey. An additional incentive is usually provided for longer surveys.

© 2021 Green AR et al. *JAMA Network Open.*

eTable 1. Comparison of Survey Respondents and US Population Benchmarks Among Adults Aged 65 Years and Older

	65+ U.S. Population Benchmarks (source: March 2019 Current Population Survey Supplement Data)		Deprescribing Communication Survey Respondents (N=835)	
Age and Sex	Frequency	Percent	Frequency	Percent
65-69 Male	8195074	15.54	145	17.37
65-69 Female	9151676	17.35	166	19.88
70-74 Male	6682348	12.67	125	14.97
70-74 Female	7434275	14.1	118	14.13
75-79 Male	4233384	8.03	79	9.46
75-79 Female	5122676	9.71	72	8.62
80+ Male	4796866	9.1	72	8.62
80+ Female	7121577	13.5	58	6.95
Race/ Ethnicity	Frequency	Percent	Frequency	Percent
White/Non-Hispanic	40180344	76.19	671	80.36
Black/Non-Hispanic	4795249	9.09	61	7.31
Other or 2+ Races/Non-Hispanic	3221462	6.11	45	5.39
Hispanic	4540821	8.61	58	6.95

© 2021 Green AR et al. *JAMA Network Open.*

eTable 2. Associations of Respondent Characteristics With Preferences for Phrases to Explain Deprescribing

eTable 2a: Statin

	**Benefits do not outweigh harms**	**No need anymore**	**Does not help anymore**	**Increased side effects**	**Extra effort**	**Harmful number of medications**	**Focus on how you feel now**
Age	-0.04 (4)	0.00 (5)	0.07 (4)	0.04 (4)	-0.02 (4)	-0.05 (4)	0.01 (4)
Prior use	-0.05 (12)	0.17 (15)	-0.04 (10)	-0.04 (11)	-0.24 (11)	0.18 (12)	-0.05 (12)
Health:							
Good	-0.04 (15)	0.25 (20)	-0.04 (14)	0.06 (15)	-0.03 (16)	-0.22 (16)	0.11 (16)
Excellent or very good	0.11 (17)	0.17 (21)	-0.08 (14)	-0.06 (16)	0.07 (17)	-0.11 (17)	-0.04 (17)
Number of medications	0.05 (6)	0.04 (7)	-0.00 (5)	-0.01 (5)	0.03 (6)	-0.12 (6)	0.03 (6)
Health literacy	0.38 (17)	-0.04 (21)	0.28 (15)	0.25 (16)	**-0.50 (17)***	-0.20 (17)	-0.27 (17)

Estimate and standard error of parameters in a linear regression of individual best-minus-worst scores on a scale of [-3, 3]. Each phrase was considered as an outcome in a separate linear regression. A negative parameter estimate means this baseline characteristic is associated with respondents choosing this phrase as less likely to make them stop taking statins. Conversely, a positive parameter estimate means the characteristic is associated with respondents choosing the phrase as more likely to make them stop taking statins. The effect is on a scale from [-3,3], where -3 means a person always chose this phrase as the least likely to make them stop taking a statin, and +3 always the most likely to make them stop taking a statin.
Age: per 5 years; reference age 65
Prior use of statins: Reference no prior use
Health: self-reported health; reference fair or poor
Number of medications: Self-reported number of medications per day, per 3 medications; reference 0 medications per day
Health literacy: High self-reported confidence in filling out medical forms (extremely or quite a bit confident); reference somewhat, a little bit, or not at all confident
* Significant at level 0.01

© 2021 Green AR et al. *JAMA Network Open.*

eTable 2b: Sedative

	More harm than good	Guidelines	Unlikely to help function	Side effects	Dependent	Without medicine	Reduce dose
Age	**-0.13 (4)***	0.09 (5)	0.08 (3)	0.00 (4)	-0.03 (4)	0.02 (5)	-0.04 (4)
Prior use	-0.06 (14)	**-0.51 (16)***	-0.3 (12)	0.02 (13)	-0.02 (15)	0.36 (16)	0.25 (14)
Health:							
Good	-0.21 (15)	0.07 (17)	-0.00 (12)	-0.20 (14)	0.01 (16)	0.20 (18)	0.17 (15)
Excellent or very good	-0.21 (16)	0.18 (18)	0.02 (13)	-0.15 (14)	-0.11 (17)	0.02 (18)	0.22 (16)
Number of medications	0.03 (5)	0.14 (6)	0.02 (4)	0.00 (5)	-0.04 (5)	-0.11 (6)	-0.05 (5)
Health literacy	-0.08 (16)	-0.15 (18)	-0.15 (13)	0.26 (15)	0.15 (17)	-0.06 (19)	-0.14 (16)
Contraindication	0.27 (11)	0.11 (12)	-0.11 (9)	0.24 (9)	-0.10 (11)	**-0.35 (12)***	-0.13 (10)

Estimate and standard error of parameters in a linear regression of individual best-minus-worst scores on a scale of [-3, 3]. Each phrase was considered as an outcome in a separate linear regression. A negative parameter estimate means this baseline characteristic is associated with respondents choosing this phrase as less likely to make them stop taking a sedative-hypnotic. Conversely, a positive parameter estimate means the characteristic is associated with respondents choosing the phrase as more likely to make them stop taking a sedative-hypnotic.
Age: per 5 years; reference age 65
Prior use of benzodiazepines: Reference no prior use
Health: self-reported health; reference fair or poor
Number of medications: Self-reported number of medications per day, per 3 medications; reference 0 medications per day
Health literacy: High self-reported confidence in filling out medical forms (extremely or quite a bit confident); reference somewhat, a little bit, or not at all confident
Contraindication: Prior fall or memory concern; reference no contraindication
* Significant at level 0.01

© 2021 Green AR et al. *JAMA Network Open.*

eTable 3. Relative Preference of Older Adults Regarding 7 Phrases a Clinician May Use to Explain Deprescribing

eTable 3a: Statin

Rationale for stopping/ reducing medicine	Mean	95% Confidence Interval
Increased risk of side effects	5.76	5.28, 6.29
Harmful number of medicines	3.82	3.52, 4.15
Benefits do not outweigh harms	3.66	3.37, 3.98
Do not need anymore	3.35	3.09, 3.64
Will not help you anymore	3.29	3.03, 3.57
Should focus on how you feel now	1.51	1.40, 1.64
Extra effort	1 (Reference)	Reference

eTable 3b: Sedative

Rationale for stopping/ reducing medicine	Mean	95% Confidence Interval
Increased risk of side effects	8.63	7.86, 9.47
Work together to reduce dose	3.98	3.67, 4.31
More harm than good	3.16	2.92, 3.42
Can become dependent	2.34	2.17, 2.53
Guidelines	2.18	2.01, 2.35
Can treat without medicines	2.03	1.88, 2.20
Unlikely to help function	1 (Reference)	Reference

© 2021 Green AR et al. *JAMA Network Open.*

eFigure. Best-Minus-Worst Score Variance

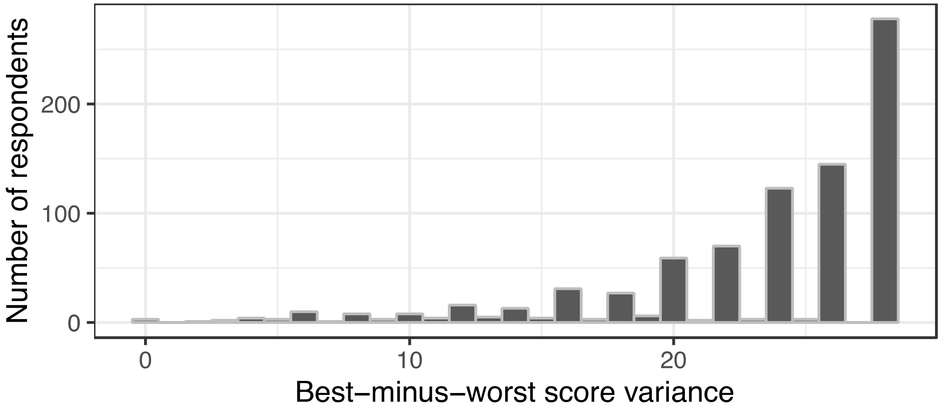

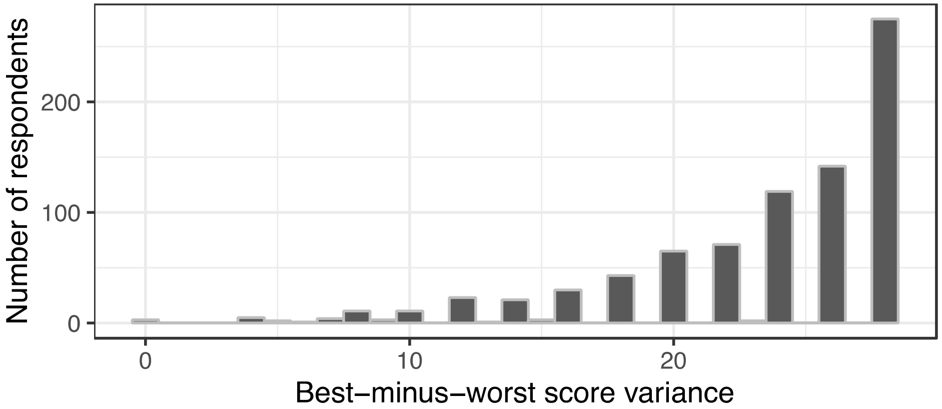

The best-minus-worst score variance is a measure of consistency of answers within each respondent. It is calculated as the sum of the squares of each individual best-minus-worst score (which here corresponds to a "most"-minus-"least" score). The higher the best-minus-worst score variance, the more consistent the answers. Most respondents' answers were moderately to highly consistent in both modules. The maximum achievable score variance was 28. With just one mistake (inadvertently confusing the two extremes), the score can drop down to 18. Few respondents did not fill out the survey and are shown here with a score variance of zero.

© 2021 Green AR et al. *JAMA Network Open.*
